# Duckweed (*Lemna minor*) as a Model Plant System for the Study of Human Microbial Pathogenesis

**DOI:** 10.1371/journal.pone.0013527

**Published:** 2010-10-25

**Authors:** Yong Zhang, Yangbo Hu, Baoyu Yang, Fang Ma, Pei Lu, Lamei Li, Chengsong Wan, Simon Rayner, Shiyun Chen

**Affiliations:** 1 State Key Laboratory of Virology, Wuhan Institute of Virology, the Chinese Academy of Sciences, Wuhan, China; 2 Department of Microbiology, School of Public Health and Tropical Medicine, Southern Medical University, Guangzhou, China; 3 Graduate School of the Chinese Academy of Sciences, Beijing, China; Fundació Institut Germans Trias i Pujol, Universitat Autònoma de Barcelona, CibeRES, Corporate Research Program on Tuberculosis (CRP-TB), Spain

## Abstract

**Background:**

Plant infection models provide certain advantages over animal models in the study of pathogenesis. However, current plant models face some limitations, e.g., plant and pathogen cannot co-culture in a contained environment. Development of such a plant model is needed to better illustrate host-pathogen interactions.

**Methodology/Principal Findings:**

We describe a novel model plant system for the study of human pathogenic bacterial infection on a large scale. This system was initiated by co-cultivation of axenic duckweed (*Lemna minor*) plants with pathogenic bacteria in 24-well polystyrene cell culture plate. Pathogenesis of bacteria to duckweed was demonstrated with *Pseudomonas aeruginosa* and *Staphylococcus aureus* as two model pathogens. *P. aeruginosa* PAO1 caused severe detriment to duckweed as judged from inhibition to frond multiplication and chlorophyll formation. Using a GFP-marked PAO1 strain, we demonstrated that bacteria colonized on both fronds and roots and formed biofilms. Virulence of PAO1 to duckweed was attenuated in its quorum sensing (QS) mutants and in recombinant strains overexpressing the QS quenching enzymes. RN4220, a virulent strain of *S. aureus*, caused severe toxicity to duckweed while an avirulent strain showed little effect. Using this system for antimicrobial chemical selection, green tea polyphenols exhibited inhibitory activity against *S. aureus* virulence. This system was further confirmed to be effective as a pathogenesis model using a number of pathogenic bacterial species.

**Conclusions/Significance:**

Our results demonstrate that duckweed can be used as a fast, inexpensive and reproducible model plant system for the study of host-pathogen interactions, could serve as an alternative choice for the study of some virulence factors, and could also potentially be used in large-scale screening for the discovery of antimicrobial chemicals.

## Introduction

The study of human diseases requires the testing of microorganisms in model systems. Traditionally, pathogenesis of human pathogens has been studied in mammalian animal models. However, experimental studies at the organism level using these mammalian models often need a large number of animals and therefore can be expensive, time-consuming, cumbersome and tedious, or ethically questionable [1]. In addition, mammalian animal models often do not fully resemble all aspects of human diseases caused by bacteria like *Pseudomonas aeruginosa* [2]. To overcome these limitations, many laboratories have turned to analyzing host-pathogen interactions using alternative animal models such as *Caenorhabditis elegans* [3]–[5], Drosophila [6], [7] and *Danio rerio* [8]. The use of these genetically tractable animal host systems have generated important information about host responses after pathogen infection and lead to a better understanding of the fundamental molecular mechanisms that underlie pathogenesis [2].

Despite the evolutionary distance between mammals and plants, both share functionally common bacterial virulence factors [2]. Mechanistic studies have proven that pathogenic bacteria also employ a similar subset of virulence determinants for attachment, secretion and genetic regulation, and ultimately to elicit disease in both animals and plants [9]–[11]. Studies have also revealed conservation of innate defense mechanisms among these diverse hosts, which suggests a potential common ancestry for these systems [12], [13]. Remarkable similarities have been uncovered in the molecular mode of pathogen-associated molecular pattern perceptions in animals and plants, including the discovery of plant receptors resembling mammalian Toll-like receptors [14]. Given these similarities and the many experimental advantages of plants, plant infection models provide a number of advantages over animal models in the study of animal pathogenesis, including ease of replication, short generation time, stringent genetic and reproductive control, and high throughput with low cost. Most importantly, using plant infection models eliminates the regulations and ethical considerations associated with using mammalian model organisms [9], [13], [15]. It has therefore been proposed that plants would make excellent models for the study of human pathogenesis [9].

Since the use of Arabidopsis to identify common themes of pathogenesis between plants and animals [10], plants have been used successfully to study pathogenesis in both Gram-negative and Gram-positive human pathogens [13], [16]. Using a clinical isolate of a *P. aeruginosa* strain and the model plant *Arabidopsis thaliana*, earlier studies have shown that this bacterium employs a similar subset of virulence factors to elicit disease in animals and plants [10], [11]. Plants have also been utilized as simple surrogate model hosts for studying host-pathogen interactions as an alternative to traditional animal pathogenesis models [17], [18]. The most widely used plant model at present for pathogen infection studies is Arabidopsis [13], [16], [18]–[20]. Research has also demonstrated that a number of human pathogens, including *P. aeruginosa* and *Staphylococcus aureus*, can also infect plants such as alfalfa [21], [22], poplar [19], lettuce [2], [13], and sweet basil [23]. However, these plant models face some limitations, e.g., the growth of plants are not in an enclosed axenic environment, plants are not uniformly developed and growth and size differences are common among individuals.

Duckweeds are monocotyledonous aquatic plants belong to the family *Araceae*. As the world's smallest, fastest growing and simplest of flowering plants, duckweeds usually reproduce by budding and can multiply very quickly in a very short amount of time. Due to their small sizes, their ability to rapid and predominantly undergo vegetative reproduction to form genetically uniform clones, easy handling and high sensitivity to organic and inorganic substances, duckweeds are widely used as model plants for studies in plant physiology, genetics, ecology and environmental monitoring [24], [25]. Given these characteristics, together with the completely artificial and enclosed environment, multiple treatments can be performed simultaneously on a large scale. We therefore propose duckweeds may be used as ideal heterologous hosts for animal pathogenic bacterial studies.

Here, we describe the development of an experimental model system using *Lemna minor* (a duckweed) as a simple and convenient host for the study of pathogenic bacterial infection. We have also demonstrated the potential of using this model system to screen anti-bacterial compounds by co-cultivation of duckweeds with pathogenic bacteria. The data presented here also demonstrate that the range of this system encompasses a variety of pathogenic bacteria, thus making it a powerful system for the study of human microbial pathogenesis.

## Materials and Methods

### Bacterial strains and growth conditions

Bacterial strains used in this study are summarized in Table 1. Strains of *P. aeruginosa* were grown in Luria–Bertani (LB) broth at 37°C with the appropriate antibiotics [26]. Strains of *S. aureus* were grown in tryptic soy broth (TSB) at 37°C [28]. Strains of *Escherichia coli*, *Salmonella enterica* Serovar Typhi, *S. enterica* Serovar Typhimurium, *Shigella dysenteriae* and *Yersinia enterocolitica* were all grown in LB broth at 37°C. *Vibrio parahaemolyticus* was grown in LB broth plus 3% NaCl at 37°C. *Y. pseudotuberculosis* strain YpIII was grown in Yersinia-Luria-Bertani (YLB) broth at 37°C with the appropriate antibiotics as described [29].

**Table 1:** Bacterial strains used in this study.

Bacterial strains	Relevant characteristics	Reference or source
***P. aeruginosa***		
PAO1	Wild type	P. Williams
PAO1-*gfp*	20 µg/ml gentamycin, PAO1 with *gfp* marker gene	P. Williams
PAO1-Δ*rhlI*	200 µg/ml tetracycline, QS *rhlI* mutant of PAO1	P. Williams
PAO1-Δ*lasI*	100 µg/ml gentamycin, QS *lasI* mutant of PAO1	P. Williams
PAO1-Δ*rhlI*/Δ*lasI*	200 µg/ml tetracycline, 100 µg/ml gentamycin, QS double mutant of PAO1	P. Williams
PAO1-pMEKm12	50 µg/ml kanamycin, PAO1 with empty vector	[26]
PAO1-*pon*1	50 µg/ml kanamycin, PAO1 with expression of human paraoxonase 1	[26]
PAO1-*pon*2	50 µg/ml kanamycin, PAO1 with expression of human paraoxonase 2	[26]
PAO1-*pon*3	50 µg/ml kanamycin, PAO1 with expression of human paraoxonase 3	[26]
PAO1-*aiiA*	50 µg/ml kanamycin, PAO1 with expression of the *aiiA* gene from *Bacillus*	[27]
***S. aureus***		
RN4220	NCTC 8325, restriction-deficient mutant, plasmid-free host strain	A. Cheung
ATCC 25923	Commonly used laboratory strain	A. Cheung
***E. coli***		
ATCC 25922	Commonly used laboratory strain	P. Williams
EHEC O157:H7 85988	Clinical isolated strain	CMCC*
**Other strains**		
*Salmonella enterica* Serovar Typhimurium 50115	Clinical isolated strain	CMCC
Serovar Typhi 50097	Clinical isolated strain	CMCC
*Yersinia enterocolitica* 52207	Clinical isolated strain	CMCC
*Y. pseudotuberculosis* YpIII	Nal^r^ wild type *Y. pseudotuberculosis*	P. Williams
*Vibrio parahaemolyticus* 20014	Clinical isolated strain	CMCC
*Shigella dysenteriae* 51252	Clinical isolated strain	CMCC

*China Medical Culture Collection.

### Establishment of duckweed-*P. aeruginosa* infection system

Duckweed (*Lemna minor*) plants were collected from the Germplasm Collection Center in Wuhan Botanic Garden, the Chinese Academy of Sciences. Axenic cultures of duckweed were initiated and maintained in 150 ml flasks containing 50 ml Schenk & Hildebrandt (SH) basal medium [30] supplemented with 1% sucrose. Flasks were kept in a growth chamber at 28°C under cool-white fluorescent lighting (90–150 µmol photons m^−2^ s^−1^) with a photoperiod of 16 h of light and 8 h of dark. Cultures were maintained by transferring 1–3 fronds into the fresh SH liquid medium at one-week intervals.

Overnight culture of *P. aeruginosa* PAO1 was harvested by centrifugation at 3000×g for 3 min, the supernatant was discarded and an equal volume of SH medium was added to resuspend the pellet, followed by centrifugation again at 3000×g for 3 min. The pellet was suspended in SH medium and adjusted to a final concentration of approximately 1×10^9^ CFU/ml. One duckweed plant was transferred into one well in sterile Costar® 24-well polystyrene cell culture cluster (Corning, USA) containing 1 ml SH medium, different volumes of PAO1 suspension were added into each well, and the total volume was adjusted to 2 ml by adding SH medium. The plates were placed in the above-mentioned growth chamber during the whole course of the experiments. Each experiment had at least six replications and at least three independent experiments were repeated to ensure validity of results. At 5 d after treatments, samples were collected for fresh weight and chlorophyll analyses as described below.

### Fresh weight and chlorophyll concentration quantification

The detrimental effects of pathogenic bacteria on duckweed were quantified by two parameters, i.e. frond fresh weight and chlorophyll concentrations. For fresh weight, at different times after co-cultivation of pathogenic bacteria with duckweed, fronds were carefully taken out by forceps, blotted dry on paper towers and the fresh weight was measured and quantified as mg fresh weight (FW)/well.

Chlorophyll concentration was determined as described [24] with modification. Briefly, after fresh weight measurement, the duckweed plants were placed into individual 1.5 ml Eppendorf tubes containing 1 ml ethanol and placed in darkness at 4°C for 24 h. Plant tissue should be completely bleached upon chlorophyll analysis. A clear solvent solution serves as the reference blank. The absorbance of the whole plant extracts is calculated from the ratio of the response of the extract solution to the reference blank according to the equations outlined by Porra *et al* [31]. The concentration in units of µg/ml of chlorophyll *a* (C*a*) is 13.70(*A*665)-5.76(*A*649) and chlorophyll *b* (C*b*) is 25.8(*A*649)-7.60(*A*665) for extraction in ethanol, and the total chlorophyll concentration was calculated as C*a*+C*b*. To mass standardize the total chlorophyll concentrations, this data was multiplied by the extraction volume (1 ml) and divided by the fresh weight of the samples (mg chl/g FW).

### GFP microscopy

Duckweeds were co-cultured with *P. aeruginosa* PAO1-*gfp* strain as described above. At different times after co-cultivation, the plants were taken out and fronds and roots were separated with forceps, placed on glass slides and observed and photographed under a fluorescence microscopy.

### Attenuated virulence of *P. aeruginosa* QS mutant and recombinant strains

*P. aeruginosa* PAO1 quorum sensing (QS) mutants of Δ*rhlI*, Δ*lasI* and Δ*rhlI*/Δ*lasI* double mutant as well as recombinant strains overexpressing the QS quenching enzymes listed in Table 1 were cultured overnight followed by centrifugation and washed as described above. 100 µl bacterial suspension of 1×10^9^ CFU/ml were added into each well containing 1 duckweed plant with a final volume of 2 ml by adding SH liquid medium. Duckweeds were collected for fresh weight and chlorophyll analyses 5 d after co-culture as described.

### Test of antimicrobial chemicals against *S. aureus*

Chitosan oligosaccharide (COS) and green tea extract (GTE) were used in this study. Both chemicals were freshly prepared before use. COS was dissolved in SH medium as 5 mg/ml stock solution and filter sterilized. GTE (purchased from the Xasino-herb Corporation, China) was dissolved in 70% ethanol at a stock concentration of 10 mg/ml. The major component of GTE was confirmed to be epigallocatechin gallate (EGCG) [32]. 100 µl *S. aureus* RN4220 of 1×10^9^ CFU/ml was inoculated into each well with 1 duckweed and different volumes of COS or GTE were then added to a final volume of 2 ml by adding SH medium. 5 days later, fresh weight and chlorophyll were quantified as described.

### Test of other pathogenic bacteria

Overnight cultures of pathogenic bacterial strains listed in Table 1 were prepared as described for *P. aeruginosa*. 100 µl bacterial suspensions were inoculated into each well and co-cultured with one duckweed plant for 5 d. Fresh weight and chlorophyll were quantified as described above.

### Data analysis

All of the experiments were replicated at least three times with at least 6 replications in each experiment. Error bars presented in the figures represent standard deviations of the means of multiple replicate experiments. The data was analyzed by one-way analysis of variance (ANOVA) (SPSS 10). A p-value of <0.05 was considered to be statistically significant.

## Results

### Virulence of *P*. *aeruginosa* to duckweed

**Figure 1:**
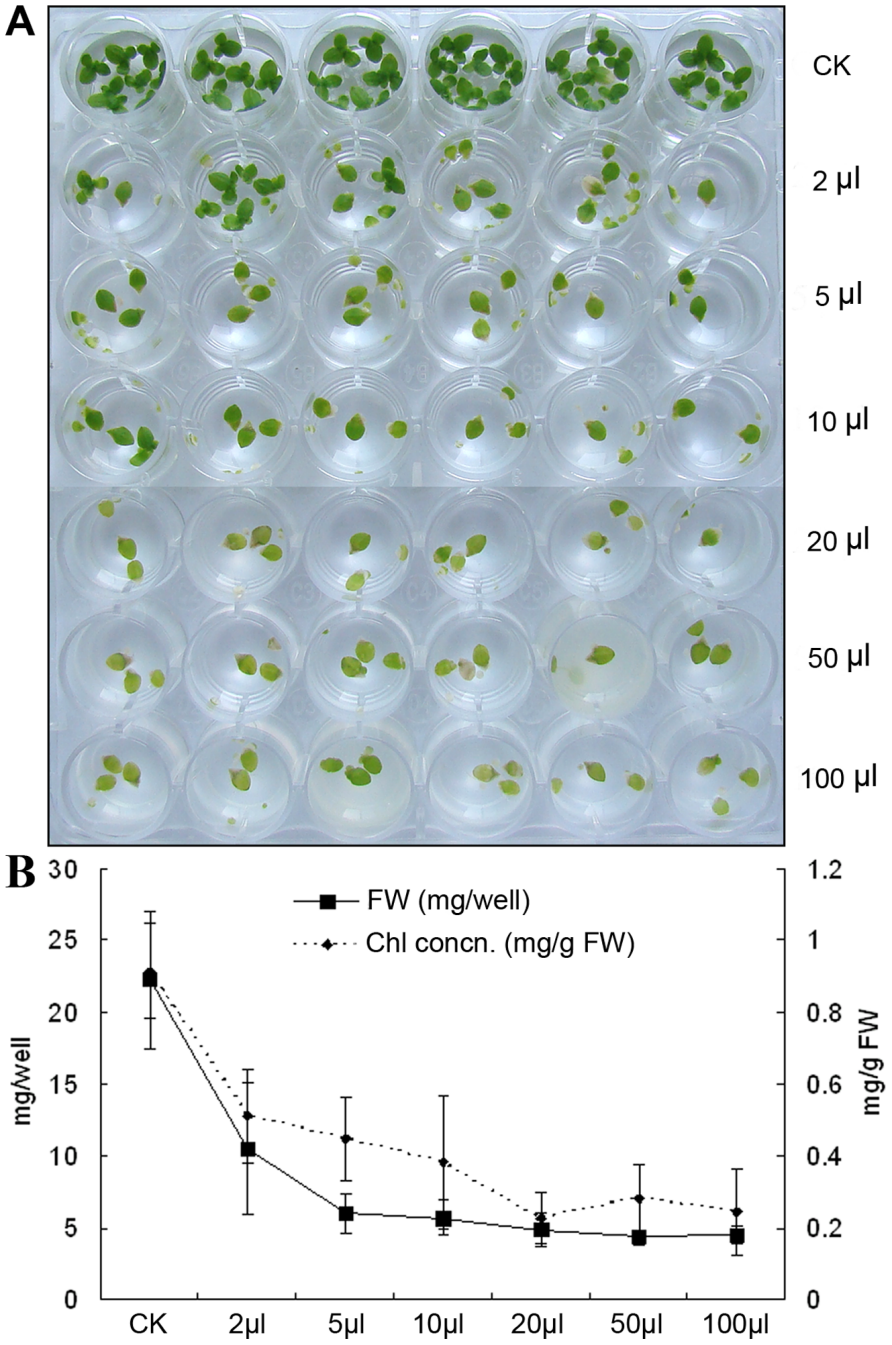
Inoculation of different volumes of *P. aeruginosa* PAO1 on duckweed growth and chlorophyll formation. A: Inhibition of duckweed growth at 5 d after inoculation of different volumes of PAO1 into the culture medium; B: Quantification by fresh weight and chlorophyll concentration.

As the first step in the process of establishing this system, a ubiquitous human opportunistic bacterium, *P. aeruginosa*, was chosen as a model pathogen. By using the procedures described in Materials and Methods, different volumes of 2 to 100 µl *P. aeruginosa* strain PAO1 suspension of 1×10^9^ CFU/ml were inoculated and co-cultivated with duckweed. Our results demonstrated that duckweed was very sensitive to the addition of *P*. *aeruginosa*; a small amount caused detrimental effects. As shown in Figure 1A, compared to the non-bacterial controls, a small amount (2 µl) inoculum of bacterial suspension was enough to elicit disease symptoms, which were characterized by collapsing of the fronds and inhibition of reproduction and growth at day 1 after inoculation, followed by chlorosis and complete maceration at 2 to 3 d after inoculation. The degree of effectiveness was subsequently quantified by fresh weight and chlorophyll concentration. The detrimental effect of PAO1 to duckweed was in a time- and dose-dependent manner. As seen in Figure 1B, the results were correlated with the amount of bacteria added as demonstrated by decreased fresh weight and chlorophyll content with the increased volumes of bacteria added.

**Figure 2:**
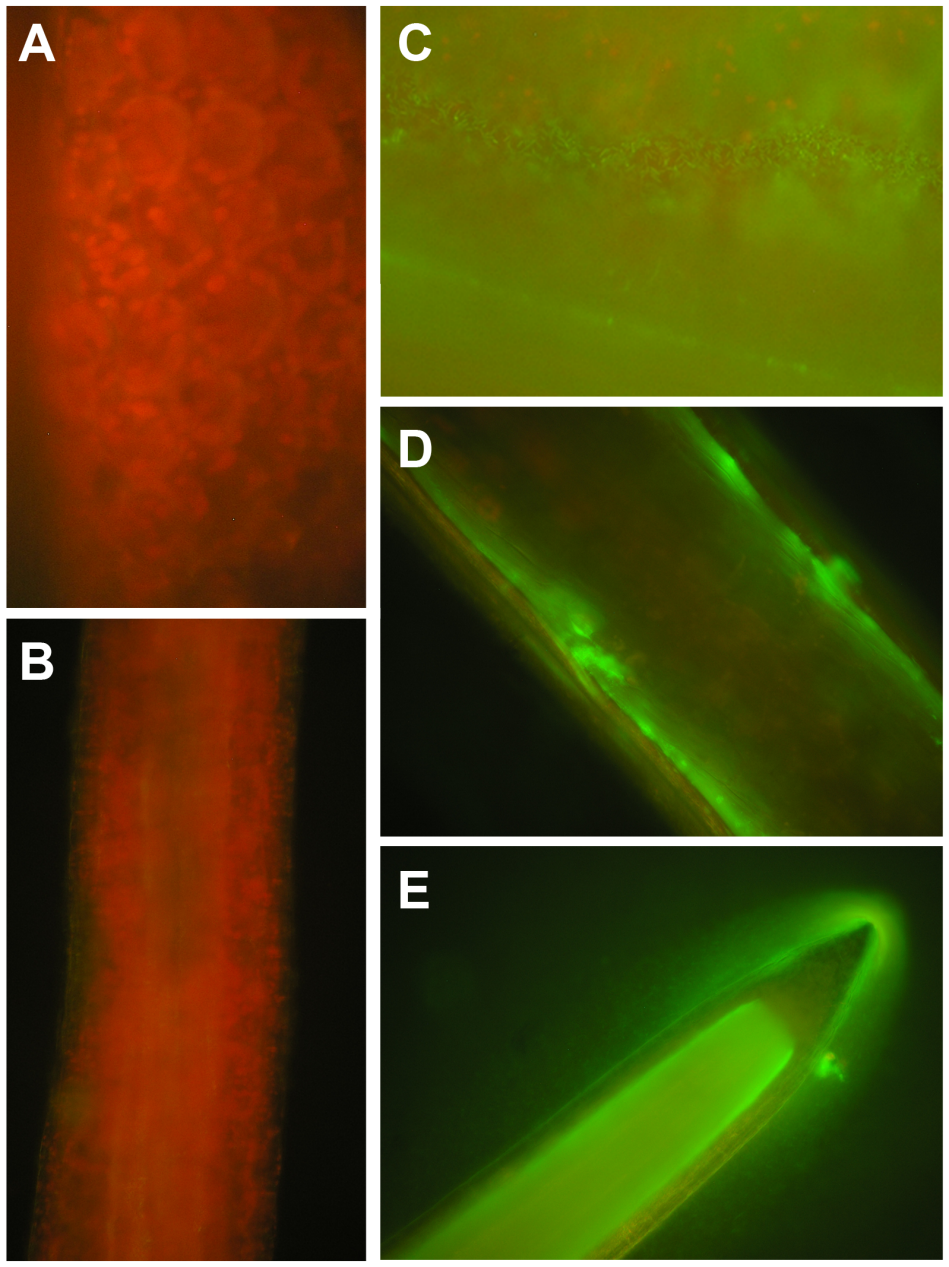
Colonization of GFP-marked *P. aeruginosa* PAO1 on duckweed frond and root under fluorescence microscopy. A & B: Control frond (A) and root (B) without inoculation of PAO1 showing autofluorescence from chlorophyll. The magnifications are 400x and 200x, respectively; C: Colonization of the frond by *P. aeruginosa* PAO1 2 d after inoculation (400x magnification); D: Colonization of the root 2 d after inoculation (200x magnification); E: Biofilm formation on the root 5 d after inoculation (100x magnification).

Having observed that *P. aeruginosa* can produce detrimental effects in duckweed, we next wanted to determine whether these effects were due to the bacterial infection. We next used a GFP-marked PAO1 strain to monitor the colonization and proliferation of bacteria on duckweed fronds and roots under a fluorescence microscope. While the control frond and root showed the typical red fluorescence caused by chlorophyll (Figure 2, A&B), the GFP-tagged bacteria could be seen on the surface of fronds and roots at the earlier stages after inoculation (Figure 2, C&D), and bacterial biofilm formation can be observed on root 5 d after inoculation (Figure 2, E).

### Attenuated virulence of quorum quenching and mutant strains to duckweed

**Figure 3:**
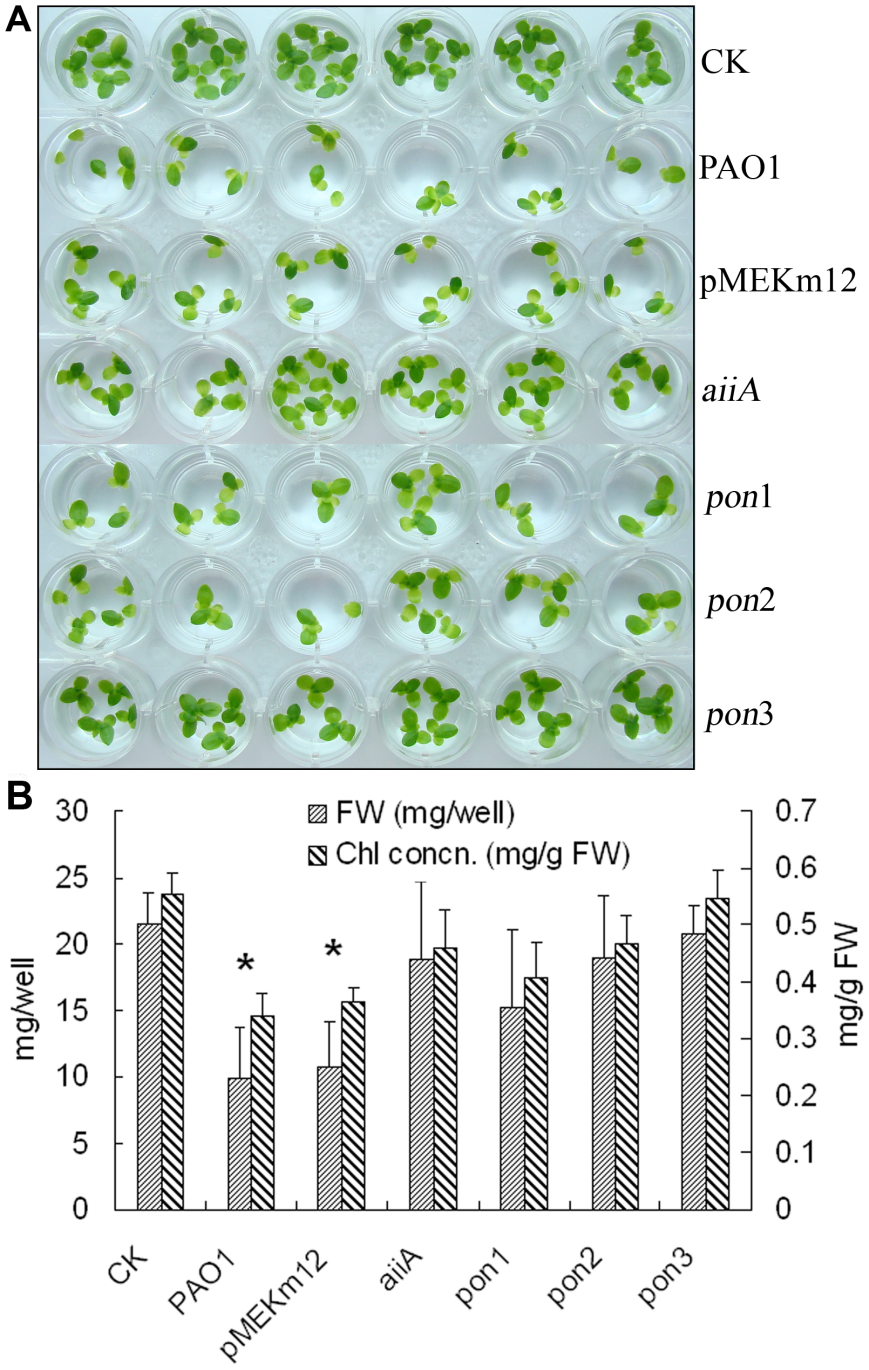
Virulence attenuation to duckweeds after co-cultivation of recombinant strains expressing the quorum quenching proteins in *P. aeruginosa*. A: Comparison of duckweed growth 5 d after inoculation of different strains as indicated on the right; B: Quantification by fresh weight and chlorophyll concentration. * P<0.05.

**Figure 4:**
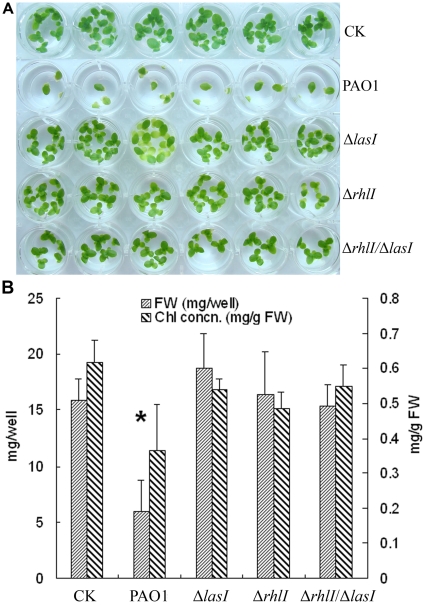
*P. aeruginosa* PAO1 QS mutants attenuated virulence to duckweed. A: Comparison of duckweed growth 5 d after inoculation of PAO1 wild type and its Δ*rhlI*, Δ*lasI* and Δ*rhlI*/Δ*lasI* double mutant strains; B: Quantification by fresh weight and chlorophyll concentration. * P<0.05.

The virulence of *P. aeruginosa* PAO1 is controlled by acyl-homoserine lactone (AHL)-dependent QS system. The human serum paraoxonases (hPONs) are a family of closely related enzymes with multiple functions, including inactivation of the QS signal molecule in *P. aeruginosa* [27], [33]. We next examined the effects of four PAO1 recombinant strains overexpressing the QS quenching enzymes (hPONs and *Bacillus* AiiA) on duckweed growth. As shown in Figure 3A, at 5 d after inoculation of these recombinant strains, duckweed growth was less affected and the virulence was attenuated compared with the wild type and empty vector controls. This was further demonstrated by the fact that the fresh weights and chlorophyll concentrations of the wells inoculated with the recombinant strains were higher than that of the PAO1 and pMEKm12/PAO1 vector control strains (Figure 3B). It is worth noting that, among the three recombinant strains overexpressing *pon1*, *pon2* and *pon3*, the attenuation effect of the strain expressing *pon3* was better than those of *pon1*and *pon2* (Figure 3A). Similarly, PAO1 QS mutant strains of Δ*rhlI*, Δ*lasI* and Δ*rhlI*/Δ*lasI* also attenuated their virulence to duckweed (Figure 4, A&B). Together, our results indicate that hPONs and AiiA expression as well as mutation of the QS-related genes result in attenuated virulence of *P*. *aeruginosa* and thus diminished pathogenicity to duckweed. It appears therefore that duckweed is a highly sensitive and effective assay system for the pathogenesis of *P. aeruginosa* strains and would serve as a model system to test virulence gene functions of pathogenic bacteria in general.

### Virulence of two *S. aureus* strains to duckweed

**Figure 5:**
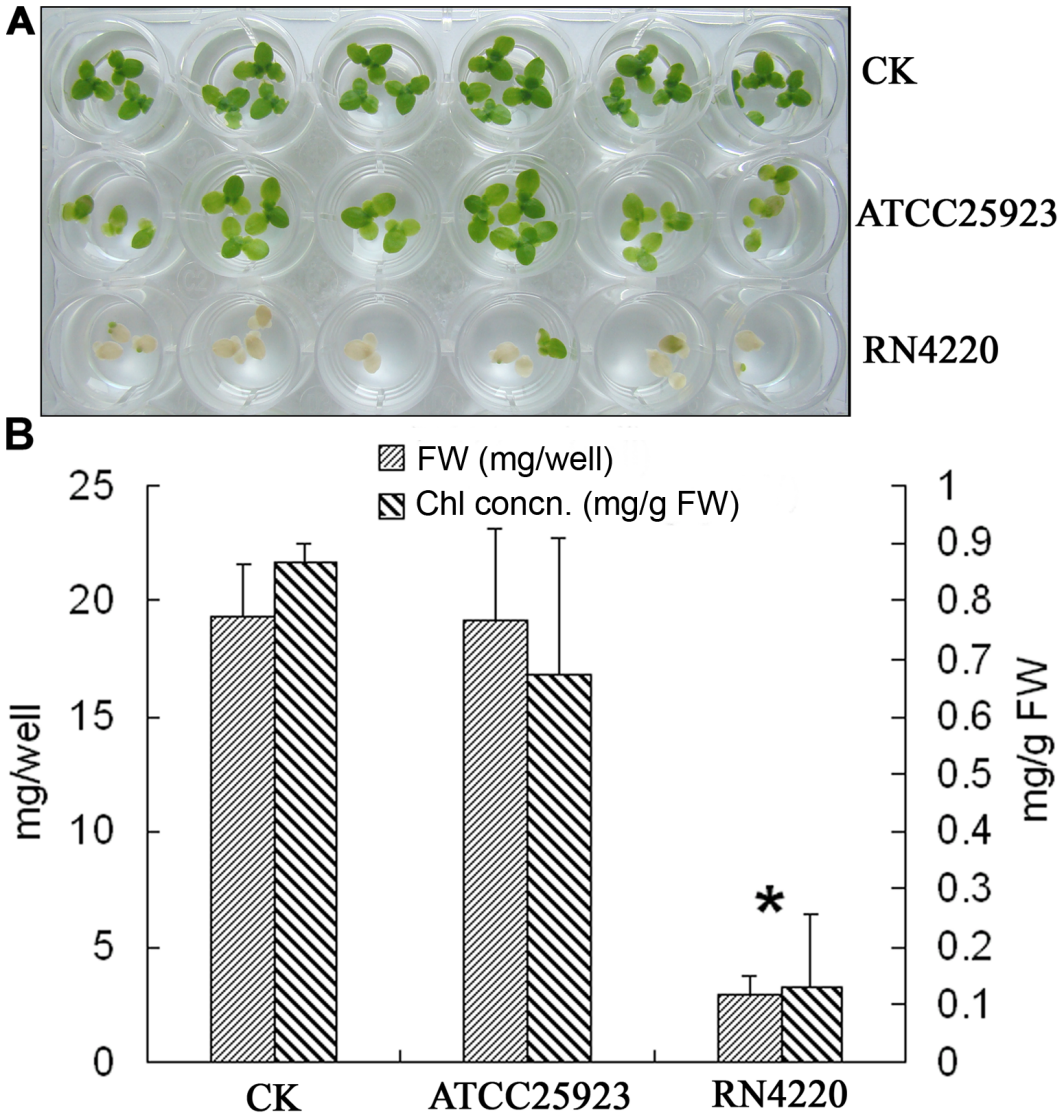
Comparison of *S. aureus* virulence strain RN4220 and avirulent strain ATCC 25923 on duckweed growth. A: Duckweed growth 3 d after inoculation of two *S. aureus* strains; B: Quantification by fresh weight and chlorophyll concentration. * P<0.05.

We next chose *S. aureus* as a Gram-positive pathogenic bacterium to further test the duckweed system. Two strains of *S. aureus* were used to test the pathogenicity to duckweed, one was the relatively avirulent strain ATCC 25923 and the other was a virulent strain RN4220 as confirmed by others [34]. As shown in Figure 5A, at 3 d after inoculation of these two *S. aureus* strains, RN4220 produced detrimental effects in duckweed as demonstrated by complete bleaching of duckweed fronds, while ATCC 25923 showed almost no difference compared with the blank control. This result was further supported by fresh weight and chlorophyll concentration data (Figure 5B). This suggests that the duckweed system can also be used to test pathogenicity of a single pathogenic bacterial species from different backgrounds in a short time. It is also worth noting that the pathogenetic symptoms to duckweed caused by *S. aureus* are different from those caused by *P. aeruginosa* (Figure 5A and Figure 1A).

### Antimicrobial chemical selection using the duckweed system

Chitosan oligosaccharide (COS) is used as an antimicrobial agent against fungi, bacteria and viruses, and its use as an elicitor of plant defense mechanisms has also been well documented [35]. We therefore chose COS to test its antimicrobial effect on *P*. *aeruginosa* and *S. aureus*. Our results showed that addition of COS into co-cultures of duckweed and bacteria did not inhibit the detrimental effects caused by either of these two bacteria in duckweed (data not shown).

**Figure 6:**
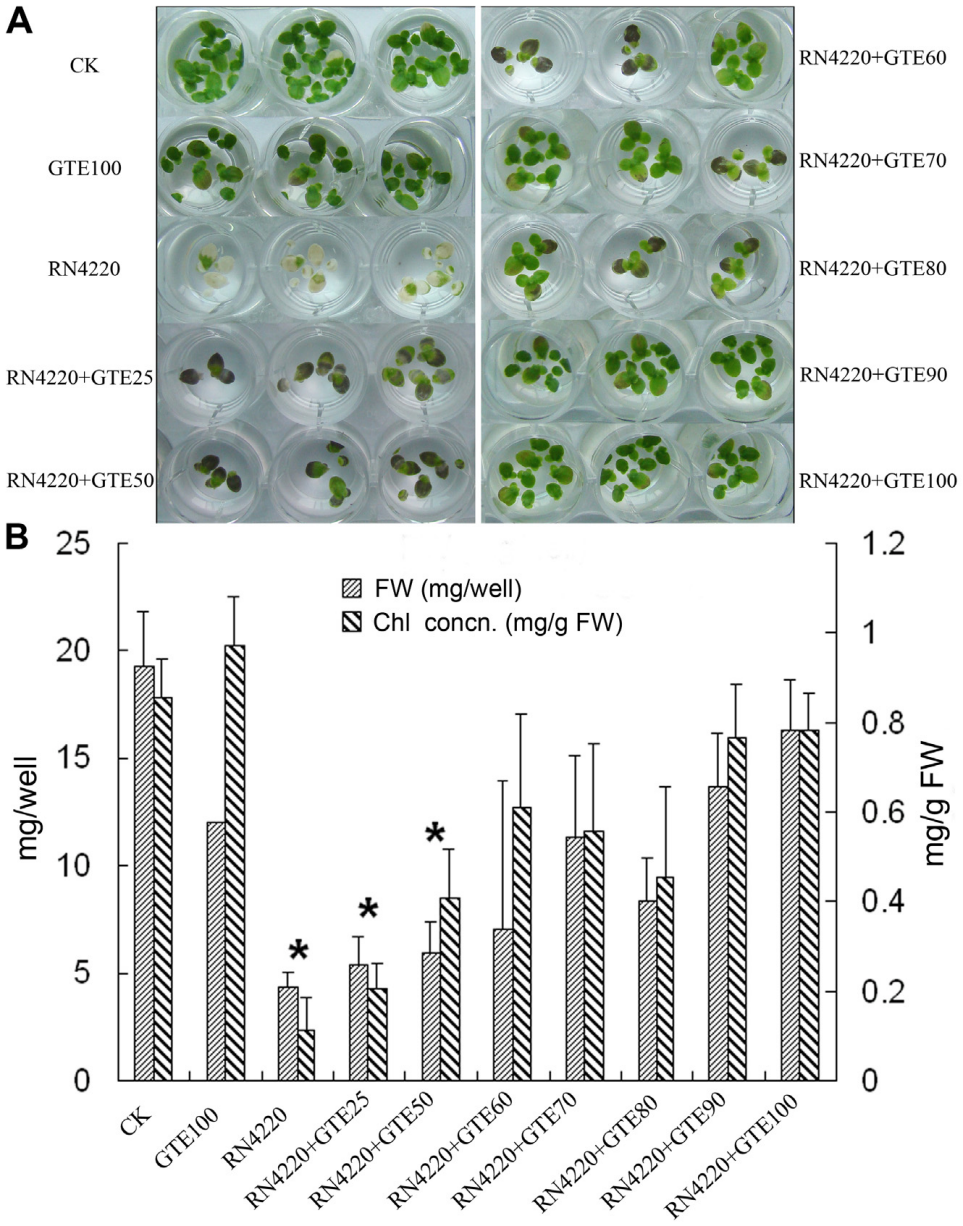
Addition of different concentrations of green tea polyphenols (GTE) on the virulence of *S. aureus* RN4220 to duckweed. A: Duckweed growth at 5 d after inoculation of RN4220 with different concentrations of green tea polyphenols; B: Quantification by fresh weight and chlorophyll concentration. * P<0.05.

It has been previously documented that EGCG, a main constituent of green tea extract (GTE), is able to act synergistically with β-lactams or carbapenems against methicillin-resistant *S. aureus* (MRSA) [36], [37]. Thus, the effect of GTE on the inhibition of *S. aureus* RN4220 virulence to duckweed was tested. As shown in Figure 6, addition of GTE attenuated *S. aureus* virulence to duckweed in a dose-dependent manner: GTE at concentrations higher than 60 mg/l inhibited *S. aureus* virulence to duckweed, while at concentrations lower than 50 mg/l *S. aureus* caused detrimental effects compared to the control. It is worth noting that, although GTE inhibited toxicity to duckweed caused by *S. aureus*, it has little toxicity on duckweed even at a concentration of 100 mg/L, indicating its potential for further development as anti-infection chemical against *S. aureus*. Together, our results demonstrate that the duckweed system could be used to screen antimicrobial compounds while at the same time show no side-effect to duckweeds.

### Test of different pathogenic bacteria on duckweed infection

**Figure 7:**
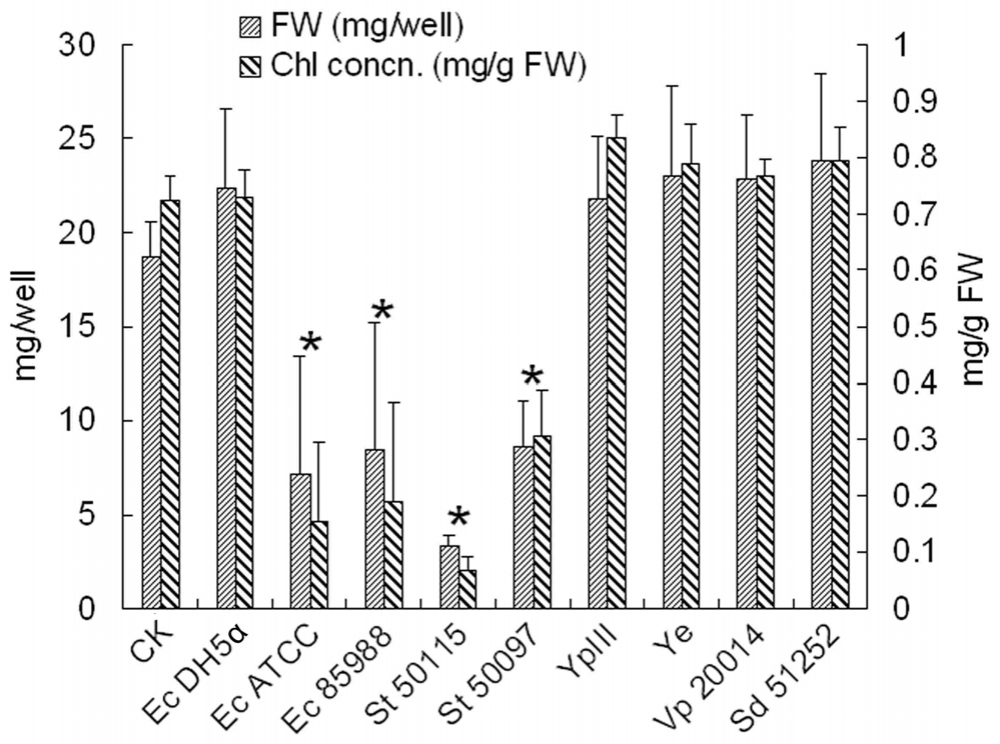
Inoculation of different pathogenic bacterial strains on duckweed growth and chlorophyll concentration. Fresh weight and chlorophyll concentration were quantified 5 d after co-cultivation of 100 µl bacterial cultures with duckweed. Please refer to Table 1 for detailed information of the numbers representing the bacterial species. * P<0.05.

We have demonstrated the effectiveness of the duckweed-pathogen system using *P. aeruginosa* and *S. aureus* as two model human pathogenic bacteria. To test if other important human pathogenic bacteria also induce pathogenicity to duckweed, a number of bacterial species representing different pathogenic backgrounds were chosen and tested. As shown in Figure 7, the severity of the symptoms varied with bacterial strain. For example, unlike the *E. coli* DH5α negative control, addition of *E. coli* ATCC 25922 and EHEC O157:H7 produced severe toxicity in duckweed, as demonstrated by complete growth inhibition and bleaching of the fronds (picture not shown). Similarly, addition of clinical isolates of *S.* Typhimurium and *S.* Typhi completely killed duckweed in one day, while strains of *Shigella dysenteriae*, *Y. pseudotuberculosis*, *Y. enterocolitica*, and *V. parahaemolyticus* showed almost no virulence to duckweed (Figure 7). In conclusion, the duckweed system is suitable for screening the virulence of large numbers of different bacterial strains in a short time.

## Discussion

Most model organisms share a set of common features that make them amenable to study in the laboratory: they are generally small, easy and inexpensive to rear in the lab, and reproduce quickly and prodigiously. In addition, model organisms are chosen on the basis that they are amenable to experimental manipulation. This usually will include characteristics such as short life-cycle, techniques for genetic manipulation and non-specialist living requirements. In this study, we have established a novel plant model system using duckweed as the host and *P. aeruginosa* and *S. aureus* as two model pathogens. Duckweed meets the criterions to be used as a model plant. Our established duckweed system for microbial pathogenesis offers many advantages over other eukaryotic systems. e.g., plants are small and grow fast, thus multiple treatments can be performed simultaneously in a contained environment with low cost. In our current study we only chose *L. minor* as the model plant. Other species of the *Araceae* family which share the similar features, e.g., *L. gibba* and *Spirodela polyrhiza*, may also be chosen as the plant host for this system.

Many common disease mechanisms are used by bacterial pathogens of plants and humans, and the interactions of bacteria with plants are often similar to the interactions of bacteria with eukaryotes [38]. Comparative analysis of alfalfa and animal models for *Burkholderia cepacia* virulence showed most of the strains that were virulent in the alfalfa infection model were also virulent in an animal model [22]. Therefore, plant models can provide fast, inexpensive and high throughput methods for discovering bacterial virulence factors [1], [39]. The duckweed system can be used to identify novel and critical pathogenicity agents, as virulence factors are often conserved across phylogeny. In addition, duckweed is a liquid-based system that is free of zoonotic pathogens and can offer greater simplicity and safety, thus is it well suited for inoculation of pathogens in the high-throughput screening of mutants of bacterial pathogens. Using a plant model, mutations in animal pathogenic bacteria can easily be screened for putative virulence factors, a process which if done using existing animal infection models would be time-consuming and tedious. Our liquid-based duckweed assay system is also ideally suited to the high-throughput screening of mutants of bacterial pathogens. QS is known to modulate many key biological functions of bacteria including virulence factors and biofilm formation [40]. Quenching QS is a promising approach to control bacterial diseases in plants and animals [41]. *lasI* and *rhlI* genes of *P. aeruginosa* PAO1 play a significant role during lung infection, mutation of these genes result in milder chronic lung infection to rat [42]. Walker *et al*. tested PAO1, *lasI* and *rhlI* mutants and the *lasI/rhlI* double mutant for pathogenicity against *Ocimum basilicum* [23]. They found that the plant responded to bacterial root infection by increasing the secretion of the antimicrobial compound rosmarinic acid from its roots. These results are in agreement with our studies using the duckweed model and can therefore be seen as a validation of the system. Since PAO1 can also form biofilm on duckweed, it can also provide a model for identifying compounds that limit biofilm formation and provides a platform for observing biofilm-deficient mutants.

Traditionally, discrimination of virulent and avirulent bacterial isolates uses specific molecular markers [43], which need expensive equipment and take time. The ability of *S. aureus* to cause a wide spectrum of disease has been attributed to its ability to produce a broad array of pathogenicity factors [4]. Using the duckweed model, virulence can be quickly assayed as demonstrated by RN4220, a strain that produces a large zone of β-hemolysin and ATCC 25923 as one relatively avirulent strain [33]. Since duckweeds were completely bleached with the addition of RN4220 at day 1 after inoculation, we harvested the plants at 3 days after co-cultivation instead of 5 days. Our results demonstrated the ability of using this duckweed system to discriminate virulent and avirulent backgrounds. This will allow us to identify a broad set of virulence factors which, in turn, may be used in the identification of novel therapeutics.

The growing problem of antibiotic-resistant bacterial pathogens points to the need for novel therapeutic approaches to combat infection; a simple infection and high-throughput screening model system is urgently needed. Using *C. elegans* as a animal model, synthetic compounds and natural product extracts that promoted nematode survival infected by two human opportunistic pathogen *Enterococcus faecalis* [44] and *P. aeruginosa* [45] have been reported. High-throughput screening using plant system can help to unravel the mechanisms by which plants resist animal pathogenic bacteria, and provides a means to discover novel therapeutic agents such as antibiotics and anti-infective compounds. It has been difficult to identify *S. aureus* virulence factors due to the unwieldiness of mammalian pathogenesis models. In our study, we confirmed the effectiveness of tea polyphenol against *S. aureus* in our duckweed model; this result needs to be tested further in a mouse model. At the same time, we also tested COS, which is effective against *Erwinia carotovora* and which has no inhibition effect on *S. aureus* virulence against duckweed. Interestingly, GTE, which is effective against *S. aureus*, has little inhibitory effect on *P*. *aeruginosa* virulence against duckweed (data not shown).

We also applied the duckweed model to examine a wider range of pathogenic bacteria; the severity of the inhibitory symptoms on duckweed depended on the bacterial strains tested. Several pathogenic bacterial species, e.g. the enteric bacteria of *Y. pseudotuberculosis* and *Y. enterocolitica*, showed no virulence to duckweed, even when the amount of bacteria doubled. This is also the case when a variety of non-pathogenic bacteria, such as *E. coli* DH5α and *Bacillus subtilis*, were used as controls (data not shown). Such differences in the pathogenicity of animal and human pathogens are known and are thought to be due to the differences in virulence as result of differential gene expression [1]. Our duckweed system made it easy to rapidly screen pathogenecity of a wide range of pathogens.

Non-mammalian host pathogenesis models also provide tools for elucidating host responses and dissecting the fundamental molecular interactions that underlie bacterial pathogenesis. Gopalan and Ausubel [17] have established a laboratory plant system using Arabidopsis interacting with different microbes to study emergence of new host-microbe adaptations. We believe our duckweed system could also be used as an alternative choice to aid in the study of how a host responds to a variety of microbes under controlled conditions. A pathogenic model in which both the pathogen and its host are amenable to genetic manipulation can greatly facilitate the understanding of bacterial pathogenesis [13]. Genome sequencing of duckweeds is underway and *Agrobacterium*-mediated genetic transformation of duckweeds (*Lemna* and *Spirodela*) has been established [46], [47]. It would therefore be interesting to transform functional genes from pathogenic bacteria into duckweeds and then use this system to test their functions. Bacterial pathogens operate by attacking crucial intracellular pathways in their hosts. It would also be interesting to use the duckweed model to further analysis how bacterial pathogens subvert host-cell pathways, which is central to understanding infectious disease.

Being able to use plant models to study pathogenicity of human pathogens relies on the microbial capacity for cross-kingdom pathogenicity [15], [18]. Of the bacterial pathogens representing 7 different genera tested in this study, several did not show any detrimental effect on duckweed, indicating that the duckweed system is not applicable for the study of all microbial pathogens; this may due to different microbial-host interactions or virulence differences. In the current study, we only tested pathogenic bacteria which are classified as biosafety class 2. It would be worthwhile to use this system testing some highly infectious bacteria, e.g., *Y. pestis* and *Mycobacterium tuberculosis*, in a biosafety level 3 (BSL-3) laboratory.

In summary, we have described the use of the aquatic plant duckweed as a model system for assessing the virulence of human pathogenic bacteria as well as assessing the therapeutic potential of anti-bacterial compounds. This model allows for high-throughput qualitative as well as quantitative analysis of novel and critical pathogenicity genes. Such a liquid-based system is especially well suited for high throughput screening experiments to identify the virulence mutant genes of bacterial pathogens in a visible manner in a short time, as well as for the identification of potential agents for the control of pathogenic bacteria.
